# Priority Control of Agricultural and Traffic Sources of Soil Heavy Metals: An Integrated Source-Oriented Risk Assessment in the Drawdown Zone of the Danjiangkou Reservoir

**DOI:** 10.3390/toxics13121073

**Published:** 2025-12-13

**Authors:** Houkuan Ding, Dahai Zeng, Yunni Gao, Xucong Lyu, Jialin Jin, Huatao Yuan, Jingxiao Zhang, Jing Dong, Xiaofei Gao, Penghui Zhu, Xuejun Li, Michele Burford

**Affiliations:** 1 College of Fisheries, Henan Normal University, Xinxiang 453007, China; 2 Observation and Research Station on Water Ecosystem in Danjiangkou Reservoir of Henan Province, Nanyang 474450, China; 3 The National Ecological Quality Comprehensive Monitoring Station (Hebi Station), Hebi 458000, China; 4 Australian Rivers Institute, Griffith University, 68 University Dr., Meadowbrook, QLD 4131, Australia

**Keywords:** drawdown zone, land-use types, heavy metals, ecological risks, human health risks, source-oriented risks

## Abstract

In recent years, the public environmental protection consciousness has improved regarding the source of drinking water. However, the risk status and sources of heavy metals (HMs) in the soil around drinking water sources remain unclear. The typical Drawdown Zone (DZ) of Danjiangkou Reservoir is taken as an example in this study. Pollution levels of HMs and associated ecological and human health risks were evaluated under four land-use types during the low-water-level period. The sources of 10 HMs were determined using the positive matrix factorization (PMF) model and correlation analysis. Quantitative source-oriented risk identification was then conducted by integrating risk characteristics with source apportionment. The results indicate that soils in the study area are generally slightly polluted, with comprehensive potential ecological risks at a medium level. Farmland soils exhibit the highest pollution and ecological risk levels, particularly for Hg and Cd. Our Monte Carlo simulation-based human health risk assessment shows that, compared with non-carcinogenic risks, carcinogenic risks should be given further attention. Farmland poses higher health risks than other land-use types, and children are more vulnerable than adults. Four main sources were identified: transportation sources (29.5%), agricultural activities (32%), natural sources (19.3%), and atmospheric deposition (19.2%). The source-oriented risk assessment indicates that agricultural activities are the priority control source for ecological risks (64.7%), with Hg as the primary control element. Transportation and agricultural sources are the primary contributors to carcinogenic risks in children (57.1%) and adults (57.1%), with Ni as the primary control element.

## 1. Introduction

The periodic artificial regulation of water levels, combined with natural climatic and topographic conditions, leads to the formation of a DZ in reservoirs [1]. Land-use patterns and anthropogenic activities within this zone significantly affect pollution loads and potential risks, thereby directly influencing reservoir water quality and ecological health [2,3]. HMs, prevalent in water, sediments and soils, are affected by both human activities and natural environmental factors [4,5,6]. Despite the importance of understanding the source-oriented risks of HMs for effective reservoir management, particularly in reservoirs that are sources of drinking water, there remains a lack of comprehensive studies integrating the multiple environmental risks of HMs with their source apportionment across common land-use patterns within the DZ.

To quantify the multiple environmental risks of a range of HMs in soils, many researchers have applied methods such as the Nemerow integrated pollution index (NIPI), the potential ecological risk index (RI), and human health risk assessment models, thereby providing essential data for evaluating ecological quality [7,8,9]. Traditional health risk assessments often rely on fixed parameter values [10], which are typically derived from limited survey data and may not accurately reflect real-world variability, thereby affecting the reliability of results [11]. Monte Carlo simulation addresses this limitation by introducing probability distributions for parameters, improving the accuracy of health risk estimates; this technique is broadly adopted [12,13]. Nonetheless, one-dimensional Monte Carlo simulation (1D-MCS) cannot fully account for parameter uncertainty, limiting its ability to reflect actual risks [14]. Specifically, there are deviations in the risk characterization of sensitive groups such as children, making it difficult to screen out the key parameters in the model to provide references for future risk control [15]. Two-dimensional Monte Carlo simulation (2D-MCS) addresses this issue through nested internal and external parameter loops [16], allowing for the verification of 1D-MCS results and the determination of the influence thresholds of the most critical parameters. This improved method has occasionally been applied in soil and groundwater risk assessments. However, there are still few studies that have applied it to source-oriented risk assessment.

Positive matrix factorization (PMF) is an approach that offers the advantage of quantifying source contributions without requiring prior assumptions about pollution origins. In recent years, it has been widely applied in apportioning HMs sources [17,18,19]. The PMF model can be integrated with potential ecological and human health risk models to identify key sources of HMs in soils. This approach enables the identification of key sources and pollutant elements by coupling quantitative source apportionment with risk indices [20,21]. However, the source-oriented assessment of HM risks in the DZ soils of reservoirs, particularly those serving as drinking water sources, remains insufficient.

The Danjiangkou Reservoir, as the exclusive water source of the Middle Route of China’s South-to-North Water Diversion Project, plays a pivotal role in ensuring the project’s sustainability and water security [22]. Following the dam heightening in 2013 and the initiation of water transfer on 12 December 2014, the reservoir’s DZ has expanded, exhibiting a distinct pattern of summer land and winter flooding [23]. This transformation from a terrestrial ecosystem to a water–land transitional area has disrupted the original ecological balance and increased environmental vulnerability [24,25]. During the dry season, which typically spans from June to November, agricultural activities, residential use, and transportation have accelerated the accumulation of HMs in the DZ, thereby increasing the amount and pollution in the reservoirs through interactions between soils and water in the flooding season. Although there are currently studies on the HMs pollution and ecological risks of the soil around the Danjiangkou Reservoir [26,27,28], there are still deficiencies in the multiple risk assessment and source analysis of soil HMs under different land-use types in the DZ.

To inform land-use planning and management within the DZ of the Danjiangkou Reservoir, this study concentrates on the eastern region, where soil HM contamination is relatively severe [26,29]. The objectives are twofold: (1) to characterize HM pollution and ecological and human health risks in soils across diverse land-use types, including forest, grassland, farmland and flooded areas; and (2) to perform a source-oriented risk assessment that quantifies the contributions of various sources to ecological and human health risks in the reservoir’s DZ.

## 2. Materials and Methods

### 2.1. Sample Collection and Analysis

The Danjiangkou Reservoir (32°360′–33°480′ N, 110°590′–110°490′ E) is located in a subtropical semi-humid monsoon zone. It is fed by several water systems, including the Han, Dan and Laoguan Rivers, and it has a large controlled drainage area with 95,200 km^2^. The reservoir primarily consists of two major sections: the Han Reservoir Area in Hubei Province and the Dan Reservoir Area situated in Henan Province [30]. The study area is located in the typical DZ of the Dan Reservoir Area, including an important inflow tributary–the Laoguan River. The main land-use types in this DZ during the dry season are farmland, forest land, grassland, and flooded area.

**Figure 1:**
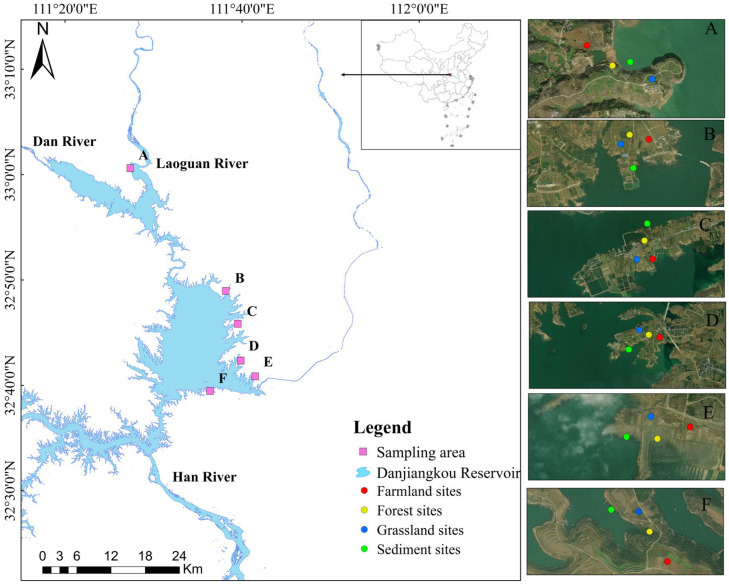
Research area and sampling sites.

Five sampling areas were selected within the DZ of Dan Reservoir, and one additional area was chosen along the Laoguan River, based on remote sensing image analysis and field investigations (Figure 1). Each sampling area includes farmland, forest land, grassland, and flooded area to ensure the collection of soil samples representing all four land-use types within every sampling location. Sampling was conducted in May, 2024, during the typical dry season, when water levels were lower than 160 m (Figure S1). To reduce the interference caused by small-scale heterogeneity and make the collected samples more representative. At each sampling site, surface soil sub-samples (0–20 cm) were collected using the five-point sampling method and mix in equal amounts to form a representative plot sample [12]. Sediments in flooded areas were obtained using a grab sampler to collect three parallel samples within a 10 m range parallel to the shoreline at each site, which were then mixed [31]. In total, 24 mixed samples were collected. After on-site removal of gravel and plant residues, the samples were placed in self-sealing bags, labeled according to the sampling area and land-use type, and then freeze-dried in the laboratory. Subsequently, the samples were ground and sieved through a 2 mm nylon mesh in preparation for the determination of ten HMs species.

The concentrations of Pb, Cu, Mn, Cr, Cd, Ni, Zn, and Fe were determined by inductively coupled plasma mass spectrometry (ICP-MS; iCAP Q, Thermo Fisher Scientific, Waltham, MA, USA) after acid digestion (HF–HNO_3_–HClO_4_). Hg and As content was measured using an atomic fluorescence spectrometer (AFS8520, Haiguang, China). Reagent blanks and standard reference materials (GBW07454, Central Plains Reference Materials Center, Henan, China) were included for quality control. Recovery rates for the reference materials ranged from 88% to 106%, and analytical errors were within ±5%, meeting the test specification requirements.

### 2.2. Pollution Measures and Potential Ecological Risk Assessment

#### 2.2.1. NIPI

The NIPI was used to assess HMs pollution in the soil [32]. The pollution index (PI) was calculated for each metal as:

(1) $$\mathrm{PI}=C_{i}/S_{i}$$

where $C_{i}$ is the measured concentration of HMs i (mg·kg^−1^) and $S_{i}$ is the background value of HMs i in the surface soil of Henan Province (mg·kg^−1^) [27]. The NIPI integrates the average and maximum PI values across all metals for each sample:

(2) $$\mathrm{NIPI}\;=\;\sqrt{\frac{\left({\mathrm{PI}}_{\mathrm{ave}}^{2}\;+{\;\mathrm{PI}}_{\max}^{2}\right)}{2}}$$

In the formula, ${\mathrm{PI}}_{\mathrm{ave}}$ and ${\mathrm{PI}}_{\max}$ are the average and maximum PI values for the metals in a sample, respectively. Classification standards for PI and NIPI are provided in Table S1.

#### 2.2.2. Potential Ecological Risk Indices

The potential ecological risk index is widely used to assess the risk of HMs in soil and sediment [33]. It includes a single-element index ($E_{r}^{i}$) and a comprehensive index (RI):

(3) $$\mathrm{RI}\;=\sum_{i=1}^{n}E_{r}^{i}=\sum_{i=1}^{n}T_{r}^{i}\;\times \;\frac{C_{i}}{B_{i}}$$

In the formula, RI represents the total ecological risk index, $E_{r}^{i}$ is the ecological risk index for metal i, $T_{r}^{i}$ is its toxicity coefficient,${\;C}_{i}$ is its measured concentration, $B_{i}$ is its background value of the element in the soil of Henan Province.

The traditional method focuses on toxicity but overlooks the combined effects of multiple metals. To address this, Men et al. [32] proposed the Nenerow Comprehensive Ecological Risk Index (NIRI), which better reflects cumulative risks:

(4) $$\mathrm{NIRI}\;=\;\sqrt{\frac{{\left(E_{r\;\mathrm{ave}}^{i}\right)}^{2}\;+\;{\left(E_{r\;\max}^{i}\right)}^{2}}{2}}$$

where the ${\;E}_{r\;\mathrm{ave}}^{i}\;$ and ${\;E}_{r\;\max}^{i}$ are the average and maximum $E_{r}^{i}$ values for a sample. The adjusted classification standards for $\;E_{r}^{i}$, RI and NIRI used in this study are shown in Table S2.

### 2.3. Human Health Risk Assessment

The health risk assessment model recommended by the US Environmental Protection Agency was applied to evaluate the carcinogenic risk (TCR) and non-carcinogenic risk (HI) of HMs in the soil for adults and children [34]. Probabilistic health risks for each HM were estimated using one-dimensional Monte Carlo simulation (1D-MCS). The formula information is shown in Text S1, and the specific parameter values and distribution types are presented in Tables S3 and S4.

Although 1D-MCS can reduce biases in traditional health risk assessment by introducing probability distributions for parameters, it does not simultaneously account for parameter uncertainty and variability [14]. Therefore, a two-dimensional Monte Carlo simulation (2D-MCS), composed of two 1D-MCS processes, was applied for verification. The structure was as follows: (1) after running 1D-MCS, the parameter with the highest variance contribution rate in the sensitivity analysis was selected as the uncertain variable and incorporated into the external loop of 2D-MCS; (2) the remaining parameters were treated as mutable variables in the internal loops. In this study, the internal loop was run for 10,000 iterations, and the external loop was set to 200 iterations, yielding 2,000,000 results from 200 probability curves. The mean of all results was used in the final analysis to ensure assessment accuracy [35].

### 2.4. Source Analysis

The sources of HMs in the soil were quantified using the PMF mode [19]. The main calculation formulae are:

(5) $$x_{\mathrm{ij}}=\underset{k=1}{\overset{p}{\sum }}g_{\mathrm{ik}}f_{\mathrm{kj}}+e_{\mathrm{ij}}$$

(6) $$Q=\sum_{i=1}^{n}\sum_{j=1}^{m}\frac{x_{\mathrm{ij}-}\overset{p}{\underset{k=1}{\sum }}g_{\mathrm{ik}}f_{\mathrm{kj}}}{u_{\mathrm{ij}}}$$

where $x_{\mathrm{ij}}$ is the concentration of the *j*-th element in the *i*-th sample; p is the number of pollution sources;${\;g}_{\mathrm{ik}}$ is the contribution of the *k*-th source to the *i*-th sample; $f_{\mathrm{kj}}\;$ is the content of the *j*-th element from the *k*-th source; $e_{\mathrm{ij}}\;$ is the residual for the *j*-th element in the *i*-th sample; and $u_{\mathrm{ij}}$ is the uncertainty of the *j*-th element in the *i*-th sample, calculated from the method detection limit (MDL), concentration, and error coefficient of the element. When the element concentration is greater than MDL:

(7) $$u_{\mathrm{ij}}=\sqrt{{\left(\mathrm{errorfraction}\;\times \;\mathrm{concentrations}\right)}^{2}\;+\;{\left(0.5\;\times \;\mathrm{MDL}\right)}^{2}}$$

When the element concentration is lower than MDL:

(8) $$u_{\mathrm{ij}}=\frac{5}{6}\times \mathrm{MDL}$$

### 2.5. Source-Oriented Risk Assessment

To quantify the potential ecological and human health risks of HMs pollutants from different sources, the PMF model was coupled with the potential ecological risk and human health risk assessment models. This approach enabled the identification of pollution sources that should be prioritized for control in the study area [36]. Details are provided in Supplementary Texts S2 and S3.

## 3. Results and Discussion

### 3.1. Characteristics of Heavy Metals Content

The mean concentrations of Pb, Cu, Mn, Ni, Zn, Cd, Cr, Hg, and As were 28.26, 34.55, 860.94, 38.02, 78.42, 0.11, 98.27, 0.05, and 12.01 mg·kg^−1^, respectively, while the mean Fe concentration was 34.30 g·kg^−1^. These average values exceeded the background levels of HMs in surface soils of Henan Province to varying degrees (Table 1). Notably, Hg exhibited the highest enrichment, reaching 2.2 times the provincial background value, indicating substantial accumulation of this element in the soil. The coefficient of variation (CV) is a measure of data dispersion, which can reflect the degree of HMs content affected by human activities to a certain extent [37]. The CV values for the 10 HMs ranked as follows: Hg > Zn > Mn > Cd > Cu > Pb > Cr > As > Ni > Fe. Fe, Ni, As, Cr, Pb, and Cu showed a moderate level of variation (16% ≤ CV < 36%), suggesting that these elements may be affected by both natural processes and human activities [11]. In contrast, Hg, Zn, Mn, and Cd exhibited high variation (CV > 36%), implying a stronger anthropogenic influence [38].

**Figure 2:**
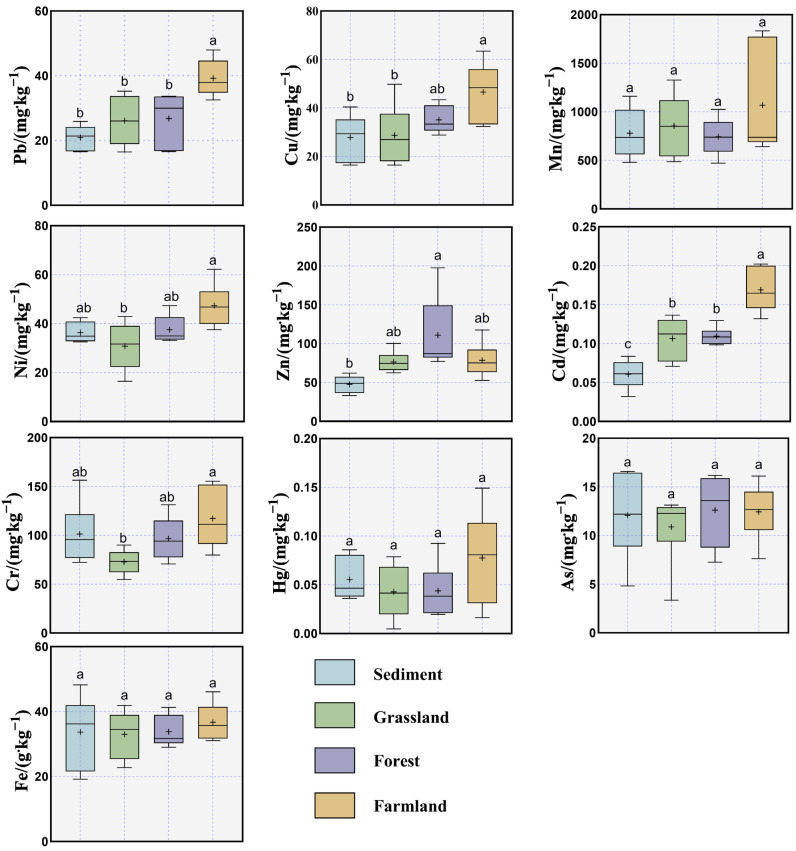
Box and whisker plots of HMs concentrations (mg·kg^−1^/g·kg^−1^) in soil from different land uses and sediment adjacent to Danjiangkou reservoir. (In the figure, a, b, and c represent the significance of differences between groups. Different letters indicate significant differences (*p* < 0.05), while the same letter indicates no significant differences).

Land use exerts marked effects on the distribution of HMs in soils [39]. The mean concentrations of Pb, Cu, Ni, Cd and Cr in farmland soils were the highest. Specifically, Pb and Cd were significantly higher than the other three land use types (*p* < 0.05), Cu was significantly higher than sediment and grassland (*p* < 0.05), and Ni and Cr were significantly higher than grassland (*p* < 0.05). The concentration of Zn in forest land was the highest, which was significantly higher than those in sediments (*p* < 0.05). The concentrations of Mn, Hg, As and Fe did not show significant differences among different land use types (Figure 2). The highest CV of Mn and Hg existed in the farmland soils. This may be attributed to variations in crop types and cultivation practices across different farmland plots. Among the six sample plots examined in this study, four were corn fields, one was a sesame field, and one was a sorghum field. Numerous studies have reported that cultivation methods and variation in crops influence the accumulation and associated risks of HMs pollution in soils [40,41]. Furthermore, we have found that the content of organic matter in the soil of agricultural land is significantly higher than that of other land types. Meanwhile, the pH content is lower than that of other land types (Figure S2). Some studies have pointed out that soil conditions with high organic matter content and low pH may lead to an increase in Hg content in the soil [42].

### 3.2. Pollution and Potential Ecological Risk Assessment

**Figure 3:**
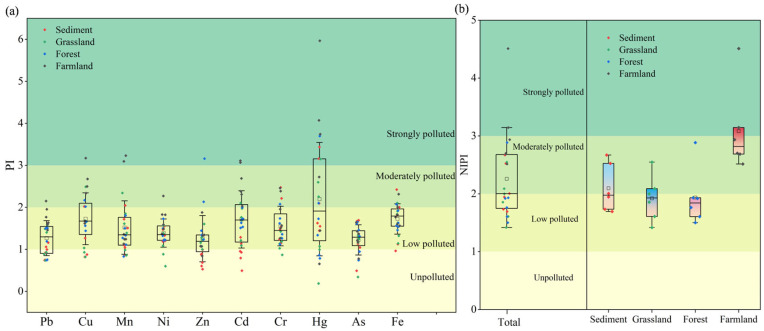
(**a**): PI indices of HMs in soils from the drawdown area. (**b**): NIPI indices of HMs in soils from the drawdown area.

The PI values of nine HMs, excluding Hg, are generally low across most sampling sites, indicating a low pollution level of HMs in the DZ of the Danjiangkou Reservoir. However, approximately half of the sampling sites exhibit moderate to strong pollution levels for Hg, as reflected by elevated PI values (Figure 3a). By integrating the average and maximum PI values across all metals for each sample, the NIPI values further indicate that most sampling sites are at a low polluted level, while a limited number of monitoring sites exhibit moderate pollution, with only a single site classified as strongly polluted (Figure 3b). Both PI and NIPI values indicated stronger pollution of HMs in farmland soils and lower pollution in forest and grassland soils. Intensive tillage and the application of chemical fertilizers and pesticides can alter soil physical and chemical properties, facilitating the accumulation of HMs [37]. This provides direct data support for our management of land-use planning in the DZ, indicating that agricultural land and non-natural agricultural activities should be reduced.

The potential ecological risk factor, $E_{r}^{i}$ results for the HMs ranked as follows: Hg (88.54) > Cd (51.79) > As (12.25) > Cu (8.64) > Ni (6.93) > Pb (6.34) > Cr (3.11) > Mn (1.51) > Zn (1.25) (Figure S3). Only Hg and Cd exceeded the thresholds for high and moderate risk, respectively; all other elements were classified as low risk. Our findings align with other studies of soils near drinking water sources, which also report high pollution levels of Hg and Cd and associated ecological risks [43,44]. This suggests that anthropogenic activities substantially contribute to the accumulation of these two elements. This risk may be significantly magnified due to the alternating dry and wet cycles in this area, forming a dynamic “source–sink” conversion process [45]. During the period when the water level of the reservoir drops, the exposed soil becomes loose due to human activities such as ploughing. When the rainy season comes or the water level rises rapidly, these fine particles carrying HMs directly enter the water body, resulting in the input of granular HMs [46]. Al-Asadi et al. [47] also pointed out that the scouring of surface runoff and the release of bottom sediment are important factors leading to the increase in HM content in water bodies. Therefore, it is necessary to pay attention to the amplification effect of water level fluctuations on the migration of heavy metals to avoid secondary pollution to water bodies.

**Figure 4:**
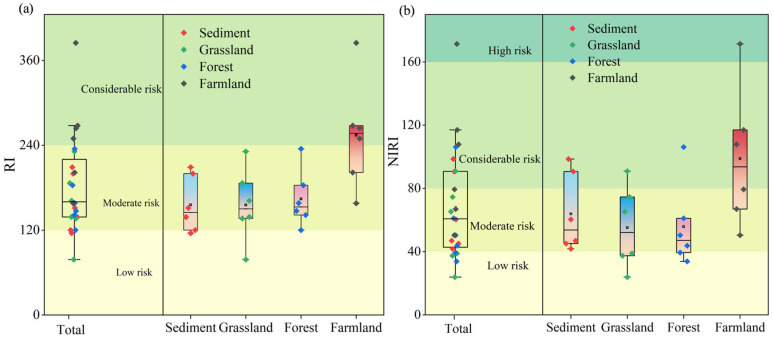
(**a**): RI indices of HMs in soils from the DZ. (**b**): NIRI indices of HMs in soils from the DZ.

The comprehensive ecological risk assessment of HMs, based on RI and NIRI values, revealed that 8% and 21% of sampling sites fell into the low ecological risks category, respectively, with 75% and 50% classified as posing moderate risks (Figure 4). A greater number of sites were classified as posing considerable and high risks based on NIRI values compared to RI values. The range of NIRI values was bigger than that of RI values. These findings indicate that the NIRI method accounts for variations arising from the number of HMs analyzed, thereby providing more accurate and comparable result [32]. However, both methods indicate that the comprehensive ecological risk of soil HMs is highest under agricultural land use.

### 3.3. Human Health Risk Assessment Based on Monte Carlo Simulation

Before conducting 2D-MCS, in order to determine the exposure parameters that are most sensitive to the health risks of different populations, we obtained the sensitivity results of each parameter based on 1D-MCS. In the 1D-MCS analysis, the soil feeding rate (IR_ing_) was identified as the most sensitive parameter affecting the health risks of adults and children (Figure S4). Therefore, when running 2D-MCS for the health risk assessment, IRing was set as the uncertain parameter for the external simulation loop, while the remaining parameters were treated as variables in the internal simulation [14]. This configuration enabled the estimation of health risks and the determination of IRing thresholds for different land-use types.

**Figure 5:**
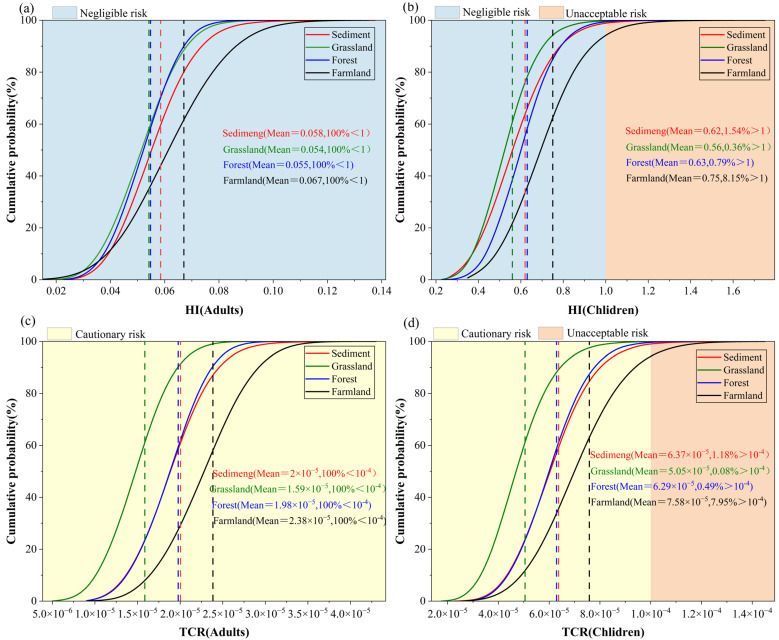
Health risk probability distribution based on 2D-MCS (**a**): Total non-carcinogenic risk for adults; (**b**): Total non-carcinogenic risk for children; (**c**): Total carcinogenic risk for adults; (**d**): Total carcinogenic risk for children. (The dotted lines represent the average risk values that correspond to each land use type).

The 2D-MCS results showed that the probability cumulative curves of the total non-carcinogenic risk index for adults did not exceed the USEPA guideline value of 1 (Figure 5a). This suggests that HM exposure through soil contact does not pose potential non-carcinogenic risks for adults in the study area [34]. For children, the overall non-carcinogenic risk remained relatively low. The probabilities of children being posed to unacceptable non-carcinogenic risks were 1.54% in sediments, 0.36% in grassland, 0.79% in forest land, and 8.15% in farmland (Figure 5b). From the perspective of carcinogenic risk, the average TCR of adults in sediments, grasslands, woodlands and farmlands is all below 10^−4^, but the probability distribution is between 10^−6^ and 10^−4^ (Figure 5c). This indicates that, although the possibility of carcinogenic risk among adults in the study area is relatively low, it deserves further attention. Among children, the risk level was increased. In total, 1.2%, 0.1%, 0.5% and 8.0% of the samples in sediment, grassland, forest and farmland, respectively, exceeded the threshold of the unacceptable carcinogenic risk category (Figure 5d).

These findings are generally consistent with the 1D-MCS results (Figure S5). Meanwhile, the results of 1D-MCS also reveal a phenomenon to us: among all land-use types, the risk index of any single HM did not exceed the warning threshold (Figures S6–S9). This indicates that the observed unacceptable risks mainly constitute the combined impact of multiple HMs rather than the influence of a single factor. However, the mean HI and TCR values for children and adults in all land-use categories were higher in the 2D-MCS than in 1D-MCS. Interestingly, the cumulative probabilities of exceeding unacceptable risk thresholds showed the opposite trend: except for HI in farmland soils, all values were lower than those obtained with 1D-MCS. This suggests that 2D-MCS produces narrower probability distribution intervals, thereby reducing the likelihood of the overestimation or underestimation of actual risks [35]. A comparison of variance (VAR), coefficient of variation (CV), and standard deviation (SD) between the two models showed that all three parameters were lower in 2D-MCS than in 1D-MCS (Table S5), further indicating that 2D-MCS improves the precision of health risk assessment [48]. Overall, 2D-MCS provides a more powerful tool for the precise assessment of health risks, especially for the sensitive group of children.

**Figure 6:**
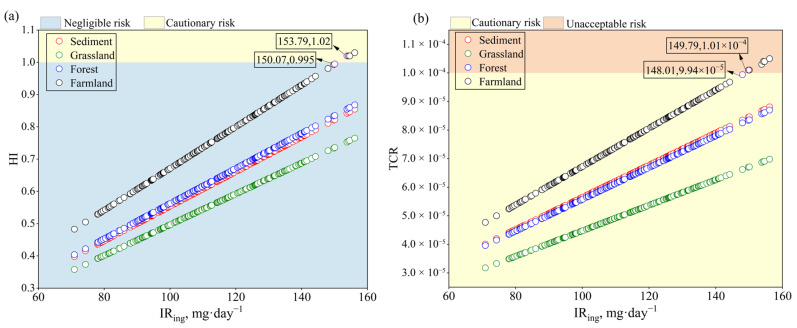
Relationship between children’s soil uptake rate and health risks under different land use types. (**a**): Total non-carcinogenic risks; (**b**): Total carcinogenic risks.

As IR_ing_ is both the most sensitive parameter in the model and the external loop variable in 2D-MCS, it is essential to evaluate health risks across different IR_ing_ values to identify critical thresholds requiring control [14]. Due to the unacceptable health risks faced by children, we chose children as a sensitive group for further research. The analysis revealed that soil ingestion within the prescribed range posed unacceptable health risks to children, but only in farmland soils. For HI, risks remained acceptable when soil ingestion was below 150.07 mg·day^−1^ but became unacceptable once it reached 153.79 mg·day^−1^. For TCR, this threshold reduced to 149.79 mg·day^−1^ (Figure 6a,b). These results provide a reference value for the maximum allowable soil ingestion to safeguard children’s health in the study area. However, it is worth noting that this conclusion is based on a specific model framework and parameter assumptions, meaning that it has certain limitations [49]. Our model treats IR_ing_ as a variable independent of other exposure parameters, while, in reality, these exposure pathways may have synergistic or offsetting effects. At the same time, the risk that heavy metals in the soil pose to the human body is mainly affected by the bioavailable forms, which may also cause deviations in the threshold of IR_ing_ [50]. Therefore, in the future, it will be necessary to consider incorporating the bioavailable forms of heavy metals into health risk assessment.

### 3.4. Source Identification of Heavy Metals

**Figure 7:**
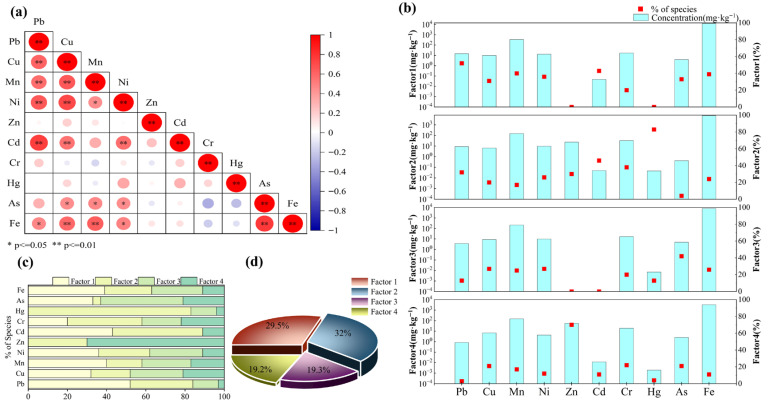
Correlation analysis of HMs in soils from the drawdown area and source apportionment using PMF analysis. (**a**): Spearman correlation analysis; (**b**): Factor Profiles; (**c**): HM factor load; (**d**): Total factor contribution.

Pearson correlation analysis and PMF model were combined to identify sources of the investigated HMs. Significant positive correlations were observed for Pb–Cu, Pb–Mn, Pb–Ni, Pb–Cd, Cu–Mn, Cu–Ni, Cu–Cd, Cu–Fe, Mn–Fe, Ni–Cd, and As–Fe (r > 0.50, *p* < 0.01, Figure 7a). This suggests that these elements may be enriched through similar geochemical processes and share a high degree of homology in origin [19]. Moderate correlations were found for Pb–Fe, Cu–As, Mn–Ni, Mn–As, Ni–As, and Ni–Fe (0.29 < *r* < 0.50, *p* < 0.05), indicating potential common sources. In contrast, Zn and Cr exhibited relatively weak correlations with other elements, implying a lower likelihood of shared sources [38].

The PMF model was applied to test multiple factor solutions and perform iterative runs. The $Q_{\mathrm{robust}}/Q_{\mathrm{ture}}$ of a four-factor solution was closest to 1 and yielded the minimum objective function Q value and optimal source profiles (Figure 7b). The residuals for most HMs were concentrated between −3 and 3, and the coefficients of determination (R^2^) between measured and predicted concentrations demonstrated strong linear relationships (Figure S10). These results confirm that the four-factor solution is reliable for the source apportionment of HMs in the soil. The contribution of each source factor and its associated HMs were identified (Figure 7c,d).

Factor 1 accounted for 29.5% of the total sources of HMs. The main loading elements were Pb (52.3%), Cd (43.4%), Mn (40.3%), Fe (39.4%), Ni (35.7%), and Cu (31%). Previous studies have reported that vehicles and other modes of transportation release substantial quantities of HMs throughout their life cycle, from production and use to disposal [51]. Pb, a characteristic pollutant from transportation, is released via fuel combustion, brake pad wear, and the degradation of lead-based coatings [52]. Cu, Ni, and Fe are widely used in brake pads and engine components due to their high thermal conductivity [53,54]. Mn is often added to fuel as methylcyclopentadienyl manganese tricarbonyl (MMT) to increase octane rating, and tire wear can release Mn-containing particles into the surrounding soil [55]. Although these metals are also present in industrial emissions, the Danjiangkou Reservoir is a water source protection zone where strict environmental controls have substantially restricted industrial activities [56]. Given the high density of roads and bridges in the study area and the frequent passage of transport vehicles serving water processing enterprises, combined with the steep terrain that increases wear of mechanical components, transportation activities are likely to be a major contributor [57]. Furthermore, the operation of ships docked in the reservoir area may also release HMs [58]. Based on these observations, Factor 1 was identified as the transportation source.

Factor 2 contributed 32% of the total, with Hg (83.3%) as the dominant loading element, and Cd (45.7%) and Cr (38.2%) also showing high loadings. As noted earlier, the coefficients of variation for Cd and Cr were 40% and 30%, respectively, while Hg reached 62%—more than 2.2 times the background value for Henan Province soils—indicating strong anthropogenic influence. Previous studies have shown that agricultural activities are major sources of Hg, Cd, and Cr in soils, as these metals are often added through fertilizers, pesticides, and insecticide [18,59,60]. Wang et al. [61] reported that farmland constitutes a substantial proportion of land use around the reservoir, with many villages situated along its banks—consistent with our findings. Most agricultural activities occur during periods of low-water levels, when growers apply more fertilizers to ensure crop maturity before water storage. Notably, Hg, Cd, and Cr are significantly enriched in farmland compared with other land types (Figure 2), suggesting that frequent agricultural practices have intensified their accumulation. Although the use of mercury-containing pesticides is now banned in China, their persistence in soils leads to long-term residues [62]. Therefore, Factor 2 was attributed to agricultural activities.

Factor 3 contributed 19.3% of the total HM sources, with As (42.1%) as the main loading element. The variability of As was relatively low, and its mean concentration was only slightly higher than the background level for soils in Henan Province (Table 1). The single-factor PI indicated that As was in a low-pollution or pollution-free state across all sampling sites, suggesting minimal influence from human activities. Previous studies have shown that As accumulation in soils is closely related to the formation of soil parent material, particularly in limestone-dominated areas [63,64]. The Danjiangkou Reservoir is surrounded by hills and low mountains where limestone is the predominant parent material. Over time, alternating erosion and sedimentation processes can cause As to accumulate in the soil surface layer. Based on these observations, Factor 3 was identified as a natural source, primarily controlled by soil parent material.

Factor 4 contributed 19.2% of the total HMs sources, with Zn (70.2%) as the primary loading element. Numerous studies have reported that lead–zinc mining is a major source of Zn accumulation in soils [65,66]. Although no mining operations were found near the sampling sites, Xichuan County, where the study area is located, is a resource-based county with extensive mineral extraction, including zinc and iron ores. Zinc ore mining generates large amounts of dust containing Zn and other HMs, which can be released into the atmosphere [67]. In the reservoir area, enhanced air convection facilitates the transport of this dust, which subsequently deposits onto the soil surface through dry and wet atmospheric deposition [68]. In addition, Zn concentrations in forest land were higher than in other land use types. The extensive canopy and large surface area of forest land favor the capture and retention of airborne particulates, including Zn-bearing particles, while limited human disturbance allows deposited Zn to accumulate over time [69]. Therefore, Factor 4 was attributed to atmospheric deposition.

### 3.5. Source-Oriented Risk Assessment

**Figure 8:**
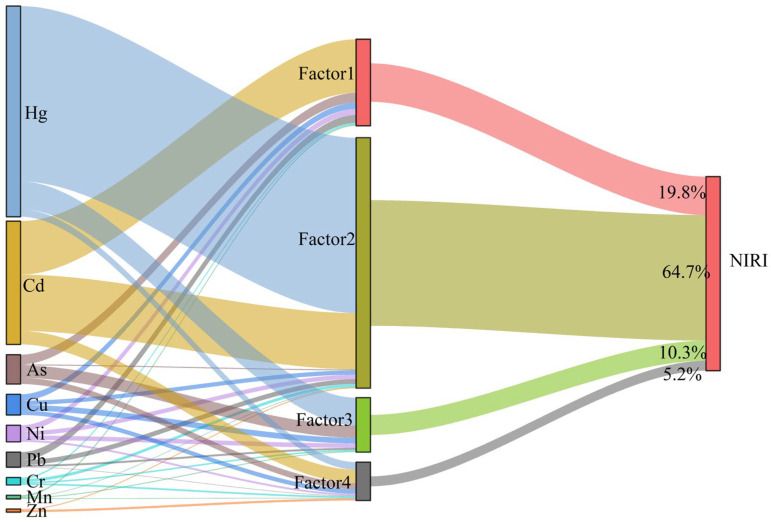
Relationship between soil HMs, their source factors, and NIRI.

Source-oriented risk assessment is essential for formulating precise prevention and control measures for HM pollution [70]. The ranking of contributions from different sources to the NIRI was as follows: agricultural activity sources (64.7%) > traffic emission sources (19.8%) > natural sources (10.3%) > atmospheric deposition sources (5.2%, Figure 8). As noted earlier, Hg was identified as originating primarily from agricultural activities. Pesticides and insecticides typically contain high concentrations of Hg, and intensive crop cultivation during the low-water level period in the water–land ecotone accelerates Hg accumulation in soil [60]. Therefore, agricultural activities and Hg were identified as the priority control sources and key management elements for mitigating comprehensive potential ecological risks in the study area.

**Figure 9:**
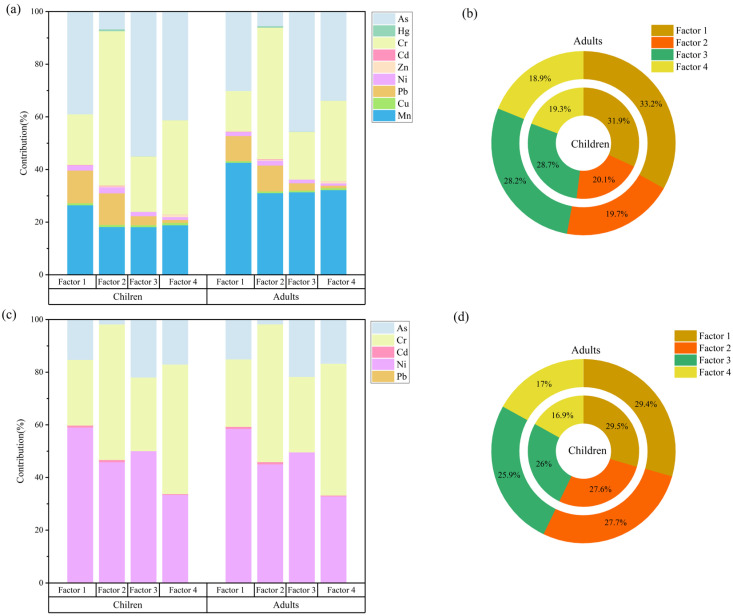
Relationship between soil HMs, their sources, and health risks. (**a**,**b**): PMF–HI; (**c**,**d**): PMF–TCR).

By integrating the human health risk assessment model with the PMF receptor model, we evaluated the risk weights of each element for HI and TCR from different sources (Figure 9a,c) and determined the source composition for HI and TCR (Figure 9b,d), in order to identify the major controlling factors for human health risks. The results indicated that anthropogenic activities including transportation and agricultural activities contributed over 50% of non-carcinogenic and carcinogenic risks for both children and adults. The main contributing element of non-carcinogenic and carcinogenic risks was As and Ni, respectively. Given the high non-carcinogenic slope factor of As, its presence increases the likelihood of non-carcinogenic effects [62]. In this study, Ni emerged as a major contributor to carcinogenic risk for both adults and children. Shen et al. [63] similarly identified Ni as the dominant contributor to carcinogenic risk in a soil HMs risk assessment for Shanghai. However, some studies have not identified Ni as a key carcinogenic risk factor, possibly due to variations in the parameter values used for risk assessment. The parameter values of the health risk assessment model in this study rely on previous research reports. In the future, these parameters should be refined based on the specific demographic characteristics of the research area. Although the primary sources of non-carcinogenic and carcinogenic risks were consistent, the key contributing elements differed. This difference is likely attributable to the varying effects of HMs on human health and to differences in reference doses and slope factors in the model [12].

## 4. Conclusions

This study evaluated the pollution levels and ecological and health risks of 10 soil HMs under different land use types in a typical DZ of the Danjiangkou Reservoir and conducted quantitative source-oriented risk identification. The pollution levels and ecological and human health risks of HMs in farmland soils were obviously higher than those in soils from forest, grassland and flooded area.

The evaluation results based on PI and NIPI indicate that only a limited number of sites exhibit moderate to strong pollution levels, with mercury (Hg) being the primary contributing contaminant.

The ecological risk assessment revealed that more than 50% of sampling sites fell into the moderate to high ecological risk categories, with agricultural-derived Hg accounting for 64.7%.

Human health risks remain mostly below safe thresholds, with higher risks to children than to adults. Anthropogenic activities (transportation and agricultural activities) contributed over 50% of non-carcinogenic and carcinogenic risks for both children and adults. As and Ni were identified as the key control elements for non-carcinogenic and carcinogenic risks, respectively.

Overall, this study established a clear source-health risk assessment framework, enhancing the accuracy of health risks. The findings highlight the need for stricter regulation and management of agricultural activities and transportation around drinking water sources to mitigate soil HMs risks in the reservoir area. At the same time, it is highly necessary to incorporate the different occurrence forms of heavy metals into the framework of risk assessment in future practices.

## Figures and Tables

**Table 1:** Descriptive statistics of HM concentrations (except for Fe, which is in g·kg^−1^; all other units are mg·kg^−1^).

Term	Pb	Cu	Mn	Ni	Zn	Cd	Cr	Hg	As	Fe
Mean	28.26	34.55	860.94	38.02	78.42	0.11	98.27	0.05	12.01	34.30
Max	47.97	63.43	1832.11	62.25	197.57	0.20	156.37	0.15	16.59	48.24
Min	16.44	16.44	471.38	16.44	33.06	0.03	54.85	0.004	3.35	19.17
SD	9.29	12.32	360.16	9.21	34.34	0.04	29.74	0.03	3.52	7.19
CV	0.33	0.36	0.42	0.24	0.44	0.40	0.30	0.62	0.29	0.21
BV	22.3	20	567	27.4	62.5	0.065	63.2	0.025	9.8	19.9

Abbreviations: SD, standard deviation; CV, coefficient of variation; BV, background values for the soil environment in Henan.

## Data Availability

The raw data supporting the conclusions of this article will be made available by the authors on request.

## Supplementary Materials

The following supporting information can be downloaded at: https://www.mdpi.com/article/10.3390/toxics13121073/s1, Text S1: Health risk assessment; Text S2: Source-oriented potential ecological risk assessment; Text S3: Source-oriented health risk assessment; Table S1: The classification of PI and NIPI; Table S2: Grading standards of potential ecological risk; Table S3: Parameter values of the health risk model based on Monte Carlo; Table S4: Reference dose and slope factor values; Table S5: Comparison of Performance Evaluation of Health Risk Assessment Models; Figure S1: Hydrological Overview of Danjiangkou Reservoir; Figure S2: Statistics of physical and chemical properties of soil; Figure S3: Potential ecological risk index of heavy metals in soils; Figure S4: Sensitivity analysis based on 1D-MCS; Figure S5: Health risk probability distribution based on 1D-MCS; Figure S6: Non-carcinogenic risk of single element in children (1D-MCS); Figure S7: Single element non-carcinogenic risk in adults (1D-MCS); Figure S8: Single Element Carcinogenic risk in children (1D-MCS); Figure S9: Single element carcinogenic risk in adults (1D-MCS); Figure S10: PMF model Fitting coefficient. (References [8,12,36,70,71,72,73] are cited in the Supplementary Materials).
