# Supplementary material for: Priority Control of Agricultural and Traffic Sources of Soil Heavy Metals: An Integrated Source-Oriented Risk Assessment in the Drawdown Zone of the Danjiangkou Reservoir
Source: Toxics. 2025 Dec 13;13(12):1073. doi: 10.3390/toxics13121073 (PMC12737257; doi:10.3390/toxics13121073)
Supplement: Supplementary file 1 [file toxics-13-01073-s001.zip › toxics-4027685-supplementary.pdf]

Supplementary materials

Priority Control of Agricultural and Traffic Sources for Soil Heavy metals: An Integrated Source-Oriented Risk Assessment in the Drawdown Zone of Danjiangkou Reservoir

oukuan Ding [1,2,3], Dahai Zeng [1,2,3], Yunni Gao [1,2,3]*, Xucong Lv[1,2,3], Jialin Jin[1,2,3], Huatao Yuan [1,2,3], Jingxiao Zhang [1,2,3], Jing Dong [1,2,3], Xiaofei Gao [1,2,3], Penghui Zhu [1,2,3], Xuejun Li [1,2,3], Michele Burford[4]

1. College of Fisheries, Henan Normal University, Xinxiang 453007, China;
2. Observation and Research Station on Water Ecosystem in Danjiangkou Reservoir of Henan Province，Nanyang 474450, China;
3. The National Ecological Quality Comprehensive Monitoring Station (Hebi Station), Hebi 458000, China.
4. Australian Rivers Institute, Griffith University, 68 University Dr, Meadowbrook, Queensland 4131, Australia.

*Corresponding Author：Yunni Gao，E-mail: gaoyn@htu.cn

Material list

Including 18 pages, 3 text, 5 tables and 10 figures

1.Text Captions

Text S1 Health risk assessment

Text S2 Source-oriented potential ecological risk assessment

Text S3 Source-oriented health risk assessment

2. Table Captions

Table S1 The classification of PI and NIPI

Table S2 Grading standards of potential ecological risk

Table S3 Parameter values of the health risk model based on Monte Carlo

Table S4 Reference dose and slope factor values

Table S5 Comparison of Performance Evaluation of Health Risk Assessment Models

3. Figure Captions

Figure S1 Hydrological Overview of Danjiangkou Reservoir

Figure S2 Statistics of physical and chemical properties of soil

Figure S3 Potential ecological risk index (E_r^i) of heavy metals in soils

Figure S4 Sensitivity analysis based on 1D-MCS

Figure S5 Health risk probability distribution based on 1D-MCS

Figure S6 Non-carcinogenic risk of single element in children (1D-MCS)

Figure S7 Single element non-carcinogenic risk in adults (1D-MCS)

Figure S8 Single Element Carcinogenic risk in children (1D-MCS)

Figure S9 Single element carcinogenic risk in adults (1D-MCS)

Figure S10 PMF model Fitting coefficient

Text S1 Health risk assessment

In this study, The HI is the sum of the Hazard Risk Quotients (HQ) for each metal and is used to assess overall Non-Carcinogenic Risk .TCR is the sum of the carcinogenic risk entropy(CR) of As, Cd, Cr, Pb and Ni and is used to assess the overall carcinogenic risk.Where ADDing、ADDder and ADDinh represent the average daily exposure dose of soil HMs by ingestion, dermal absorption, and inhalation, respectively[12].

$$ADD_{ing}=\frac{C_i \times IR_{ing} \times EF \times ED}{BW \times AT} \times 10^{-6} \quad (1)$$

$$ADD_{der}=\frac{C_i \times SA \times SL \times ABF \times EF \times ED}{BW \times AT} \times 10^{-6} \quad (2)$$

$$ADD_{inh}=\frac{C_i \times IR_{inh} \times EF \times ED}{BW \times AT \times PEF} \quad (3)$$

$$HI=\sum HQ = \sum \frac{ADD_i}{RFD_i} \quad (4)$$

$$TCR=\sum CR=\sum ADD_i \times SF_i \tag{5}$$

The specific parameters of the above formulae were shown in Tables S3 and S4

Text S2 Source-oriented potential ecological risk assessment

The PMF model, in combination with the comprehensive ecological risk assessment index method, quantitatively analyzes the contribution rate of each pollution source to ecological risks, which can accurately assess the risks caused by heavy metal pollution sources to the ecological environment[70,71]. The formula is as follows.

$$NIRI_i^k=\sqrt{\frac{(E_{r\,ave}^{ik})^2+(E_{r\,max}^{ik})^2}{2}} \tag{6}$$

Where, $NIRI_i^k$ is the ecological risk of pollution source k to multiple elements in sample i, $E_{r\,ave}^{ik}$ and $E_{r\,max}^{ik}$ are the maximum and average ecological risk values of all elements in the same sample, respectively[8].

Text S3 Source-oriented health risk assessment

The quantification of health risks was calculated using the following formula:

$$ADD_{ing}^k=\frac{C_i^k \times IR_{ing} \times EF \times ED}{BW \times AT} \times 10^{-6} \tag{7}$$

$$ADD_{der}^k=\frac{C_i^k \times SA \times SL \times ABF \times EF \times ED}{BW \times AT} \times 10^{-6} \tag{8}$$

$$ADD_{inh}^k=\frac{C_i^k \times IR_{inh} \times EF \times ED}{BW \times AT \times PEF} \tag{9}$$

$$HI^k=\sum HQ^k=\sum \frac{ADD_i^k}{RFD_i} \tag{10}$$

$$TCR^k=\sum CR^k=\sum ADD_i^k \times SF_i \tag{11}$$

HI^k represents the sum of the HQ from the k-th source, and the TCR^k represents

the sum of the CR from the k-th source[36].

Table S1 The classification of PI and NIPI

Potential ecological risk of individual factors	Risk level	Potential ecological risk index	Risk level
PI≤1	Unpolluted	NIPI≤1	Unpolluted
1<PI≤2	Low polluted	1<NIPI≤2	Low polluted
2<PI≤3	Moderately polluted	2<NIPI≤3	Moderately polluted
PI>3	Strongly polluted	NIPI>3	Strongly polluted

Table S2 Grading standards of potential ecological risk

Potential ecological risk of individual factors	Risk level	Potential ecological risk index	Risk level	Nemerow integrated risk index	Risk level
E_r^i<40	Low risk	RI<120	Low risk	NIRI<40	Low risk
40≤E_r^i<80	Moderat-e risk	120≤RI<240	Moderate risk	40≤NIRI<80	Moderate risk
80≤E_r^i<160	Consider-able risk	240≤RI<480	Consider-able risk	80≤NIRI<160	Consider-able risk
160≤E_r^i<320	High risk	480≤RI<960	High risk	160≤NIRI<320	High risk
E_r^i≥320	Extreme risk	RI≥960	Extreme risk	NIRI≥320	Extreme risk

Table S3 Parameter values of the health risk model based on Monte Carlo[72]

Parameter	Description	Unit	Distribution type	Value
IR_{ing}	Ingestion rate of soil	mg • day^{-1}	Triangle	Children(66,103,161),Adult（40,30,52）
IR_{inh}	Inhalation rate of soil	m^3 • day^{-1}	point	Children:8.6,Adult:19

SA	Skinarea available for soil contact	m^2	point	Children:0.23,Adult:0.54
SL	Soil-to-skin adherence factor	$mg \cdot cm^{-2}$	Log-normal	Children(0.65,1.2),Adult （0.49,0.54）
ABF	Absorption factor	unitless	point	0.001(NCR),0.01(CR)
PEF	Particle emission factor	$m^3 \cdot kg^{-1}$	point	1.36×10^9
EF	Exposure frequency	$day \cdot year^{-1}$	Triangle	180,345,365
ED	Exposure duration	year	point	Children:6,Adult:24
BW	Body weight	kg	Log-normal	Children(16.68,1.48),Adult （56.4,11.9）
AT	Average time	day	point	365×ED(NCR),365×70(CR)

Table S4 Reference dose and slope factor values[73]

Element	RFD $(mg \cdot kg^{-1} \cdot day^{-1})$			SF $(mg \cdot kg^{-1} \cdot day^{-1})^{-1}$		
	RfD_{ing}	RfD_{der}	RfD_{inh}	SF_{ing}	SF_{der}	SF_{inh}
Pb	3.5×10^{-3}	5.25×10^{-4}	3.52×10^{-3}	8.5×10^{-3}	8.5×10^{-3}	4.2×10^{-2}
Cu	4×10^{-2}	1.2×10^{-2}	4.02×10^{-2}			
Mn	4.6×10^{-2}	1.84×10^{-3}	1.43×10^{-5}			
Ni	2×10^{-2}	5.4×10^{-3}	2.06×10^{-2}	1.7	4.25×10^{-1}	8.4×10^{-1}
Zn	3×10^{-1}	6×10^{-2}	3×10^{-1}			
Cd	1×10^{-3}	1×10^{-5}	1×10^{-5}	6.1	3.80×10^{-1}	6.3
Cr	3×10^{-3}	6×10^{-5}	2.86×10^{-5}	5×10^{-1}	2×10^{-1}	4.2×10^{-1}
Hg	3×10^{-4}	8.5×10^{-5}	2.1×10^{-5}			
As	3×10^{-4}	3×10^{-4}	1.23×10^{-4}	1.5	1.51×10^{-1}	3.66
Fe	——	——	——	——	——	——

Note: “—”represents the toxic parameters for metals under the corresponding exposure pathways were unavailable

Table S5 Comparison of Performance Evaluation of Health Risk Assessment Models

			Sediment	Grassland	Forest	Farmland
Standard	HI	1D-MCS	0.17	0.16	0.16	0.20

deviation		2D-MCS	0.15	0.13	0.14	0.17
	TCR	1D-MCS	5.801×10^{-6}	1.48×10^{-5}	1.683×10^{-5}	1.791×10^{-5}
		2D-MCS	1.424×10^{-5}	1.214×10^{-5}	1.292×10^{-5}	1.653×10^{-5}
Variance	HI	1D-MCS	0.03	0.03	0.03	0.04
		2D-MCS	0.02	0.02	0.02	0.03
	TCR	1D-MCS	2.087×10^{-10}	2.192×10^{-10}	$2.832\text{E}\times10^{-10}$	3.208×10^{-10}
		2D-MCS	3.366×10^{-10}	1.517×10^{-10}	1.718×10^{-10}	2.81×10^{-10}
Coefficient of variability	HI	1D-MCS	0.30	0.29	0.29	0.29
		2D-MCS	0.25	0.23	0.22	0.23
	TCR	1D-MCS	0.27	0.30	0.27	0.29
		2D-MCS	0.22	0.24	0.21	0.22

Notes:Take the assessment results of children as an example

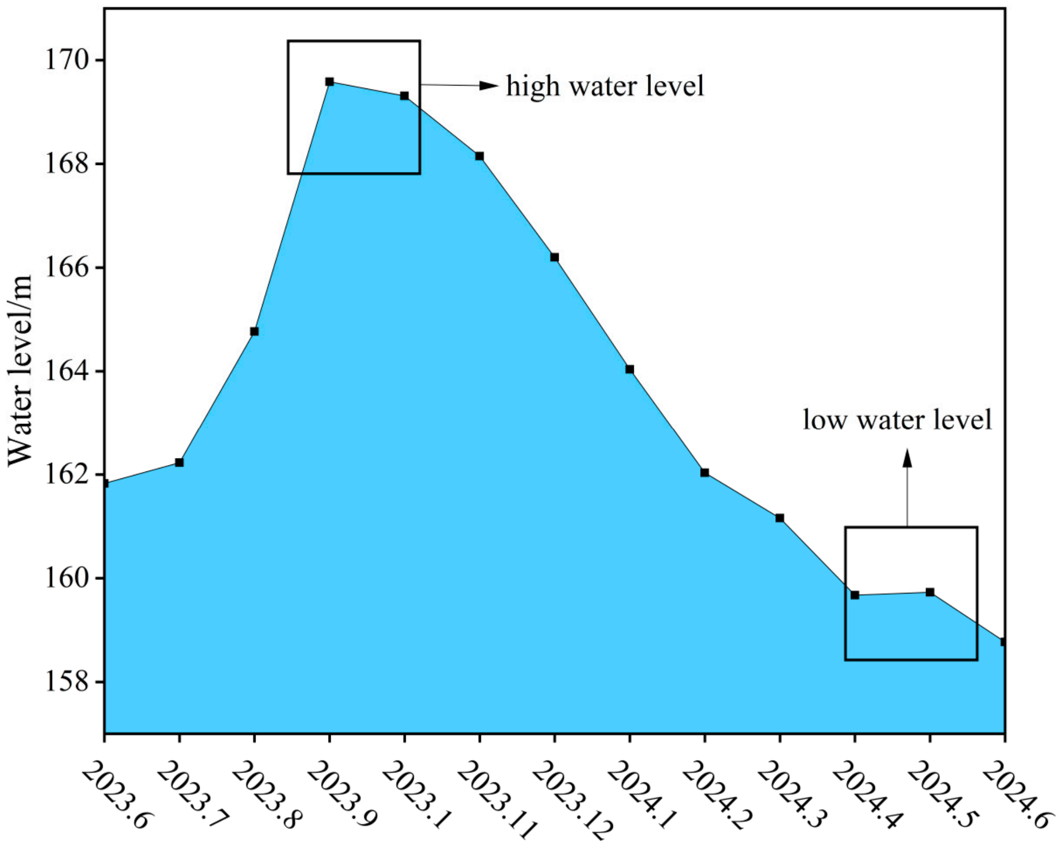

Figure S1 Hydrological Overview of Danjiangkou Reservoir

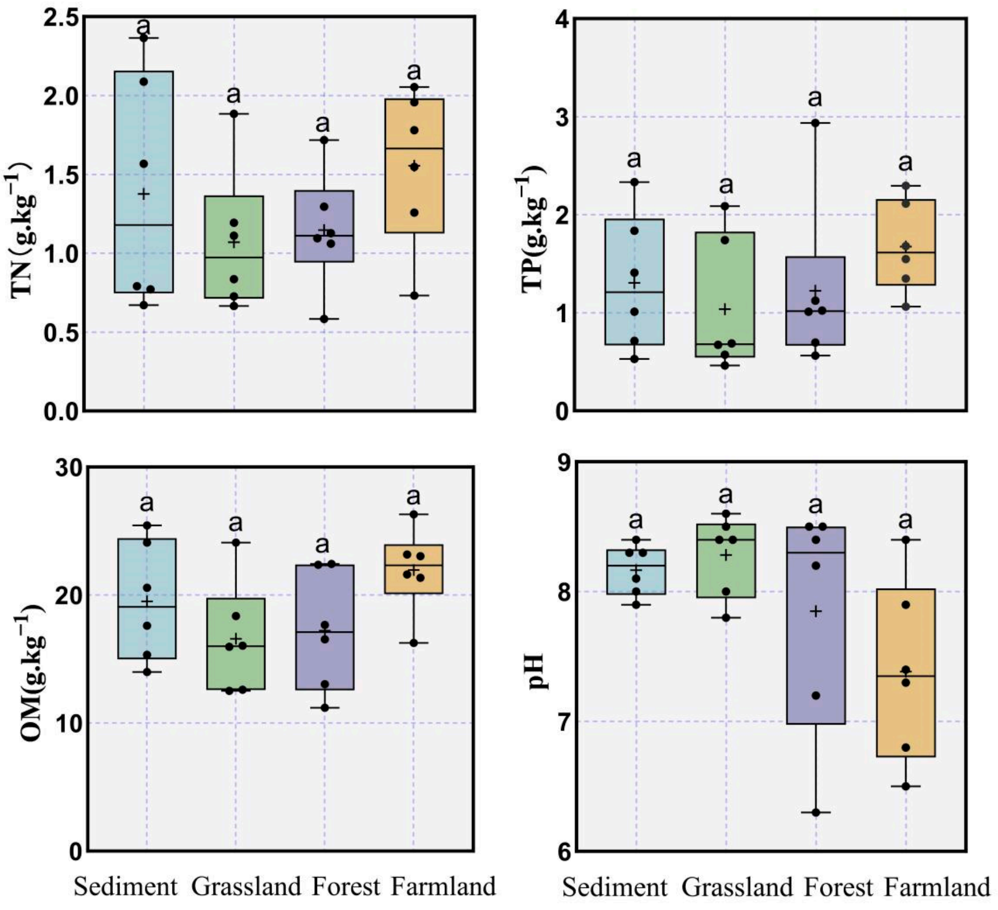

Figure S2 Statistics of physical and chemical properties of soil.(In the figure, a,b, and c represent the significance of differences between groups. Different letters indicate significant differences ($p < 0.05$), while the same letter indicates no significant differences)

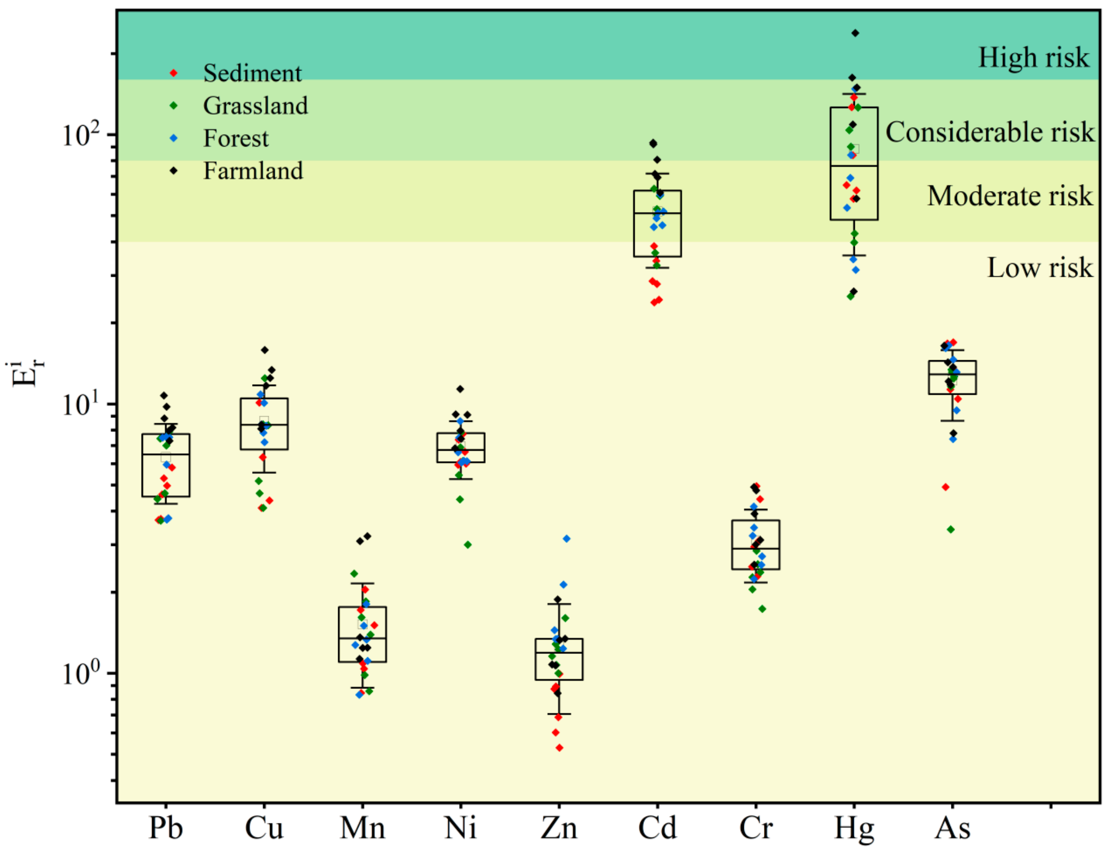

Fig S3 Potential ecological risk index (E_r^i) of heavy metals in soils

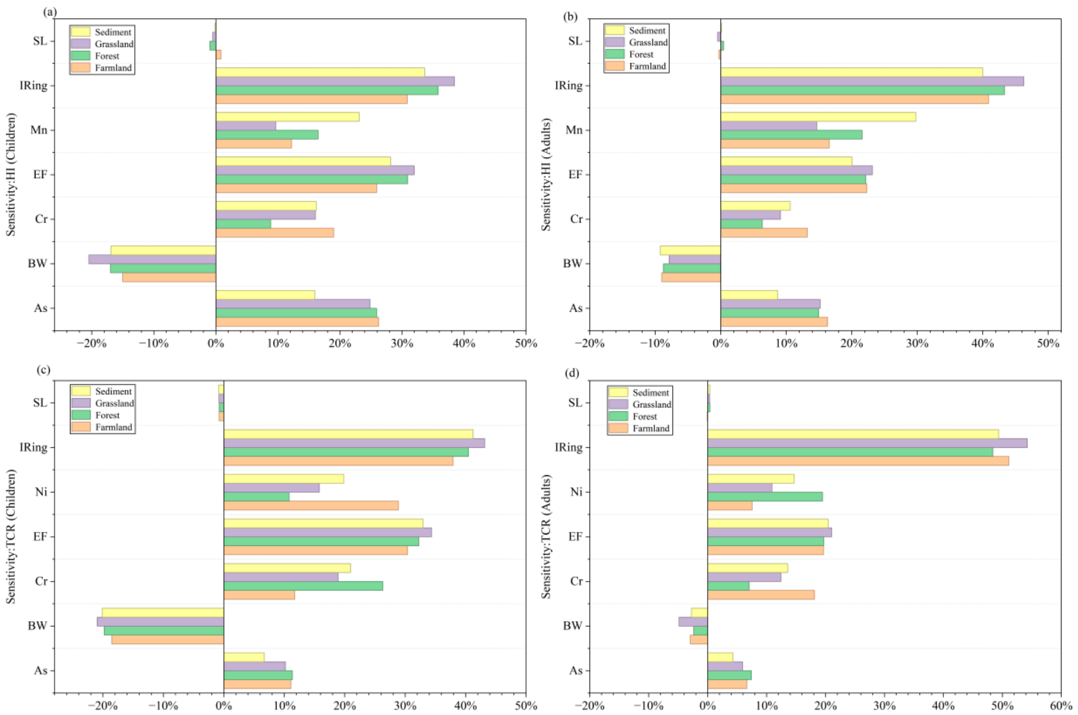

Figure S4 Sensitivity analysis based on 1D-MCS

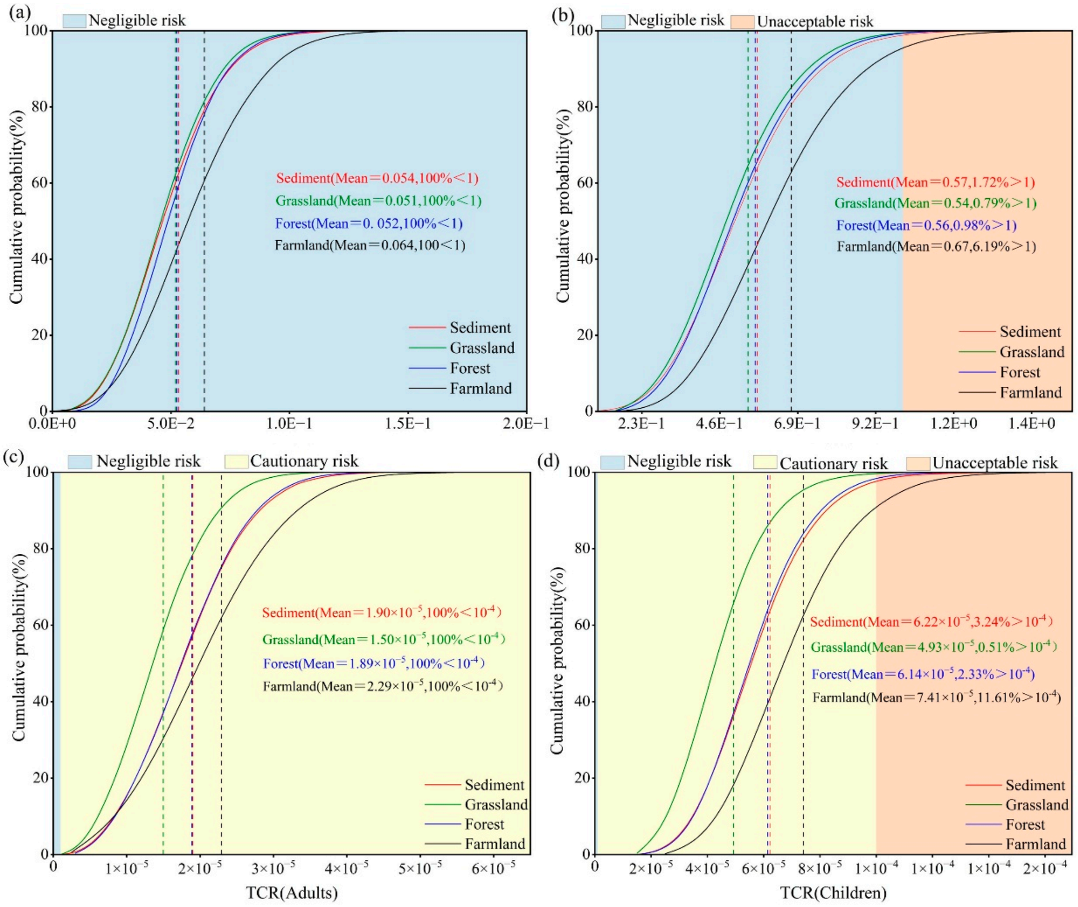

Figure S5 Health risk probability distribution based on 1D-MCS

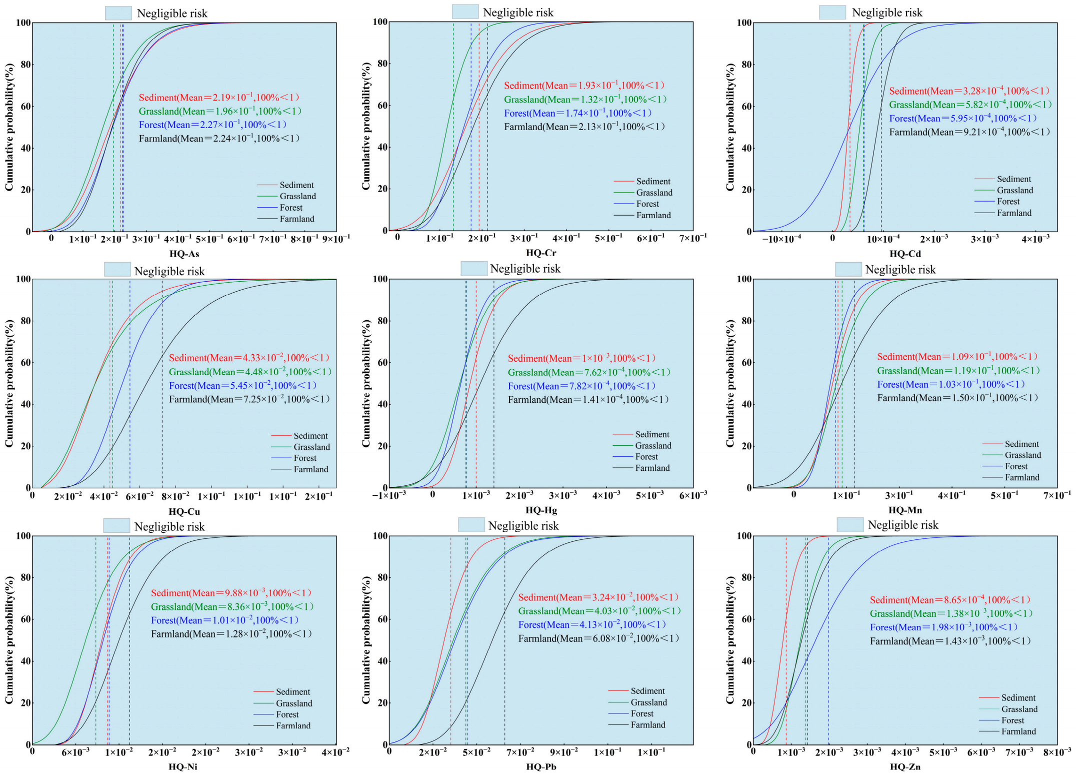

Figure S6 Non-carcinogenic risk of single element in children (1D-MCS)

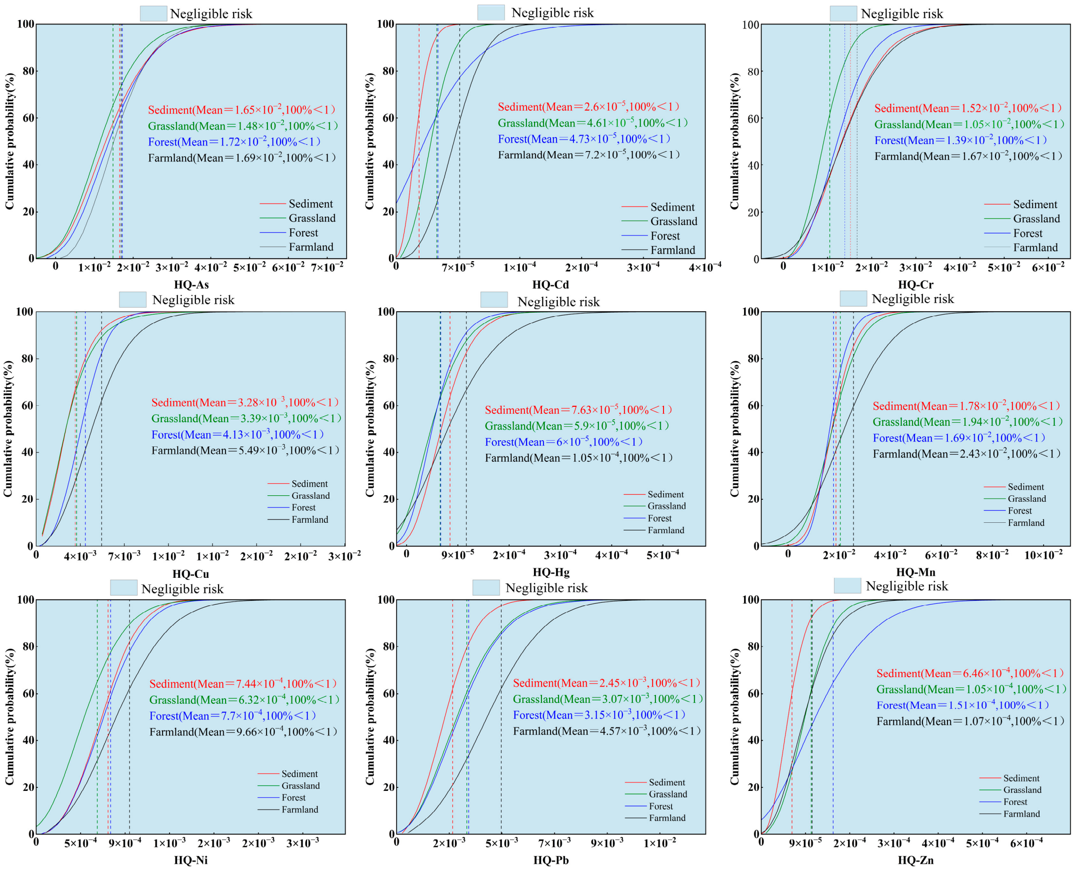

Figure S7 Single element non-carcinogenic risk in adults (1D-MCS)

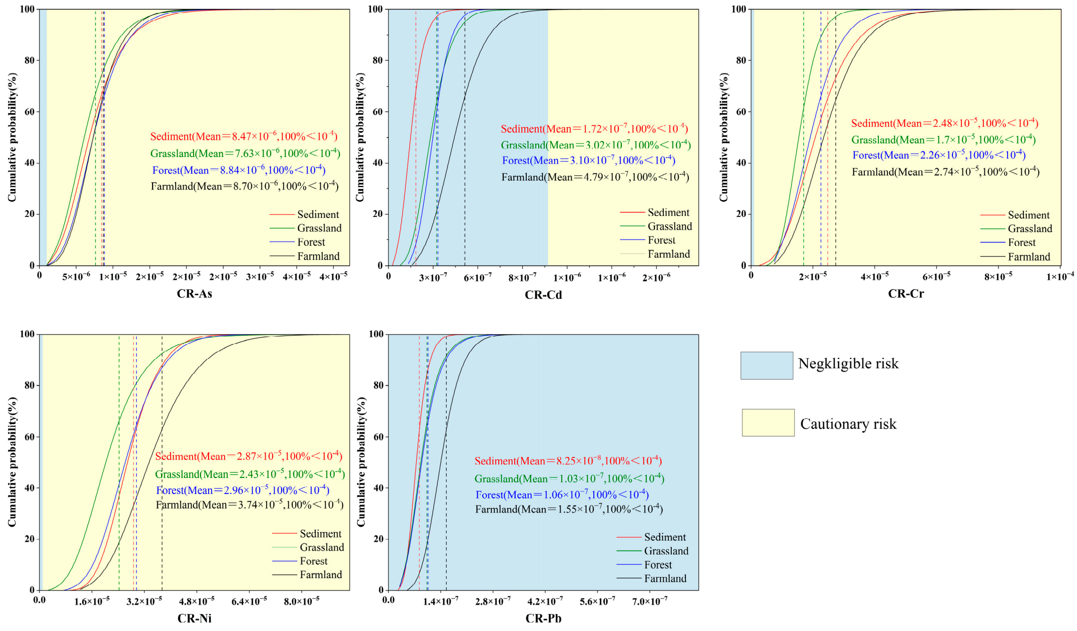

Figure S8 Single Element Carcinogenic risk in children (1D-MCS)

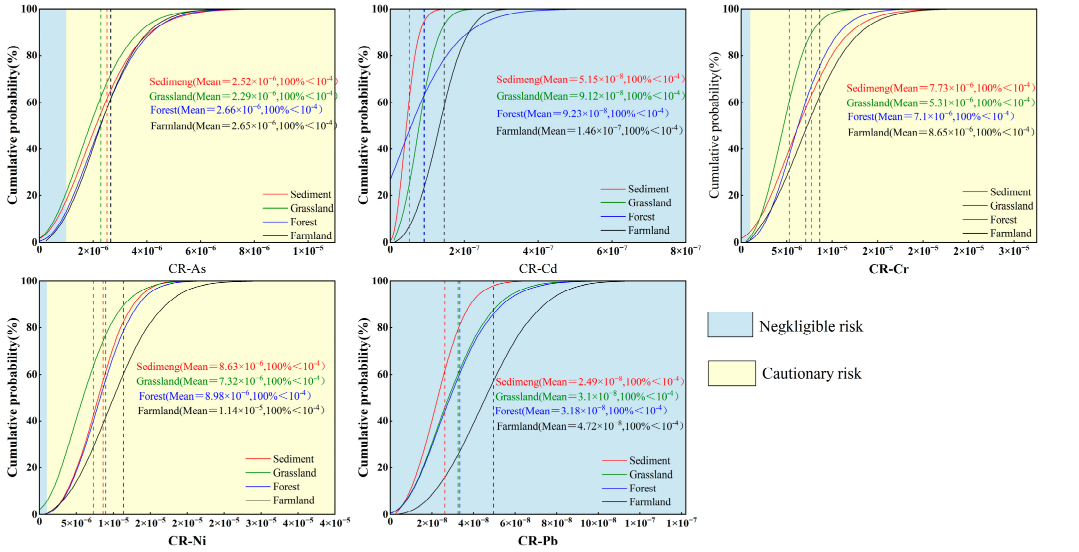

Figure S9 Single element carcinogenic risk in adults (1D-MCS)

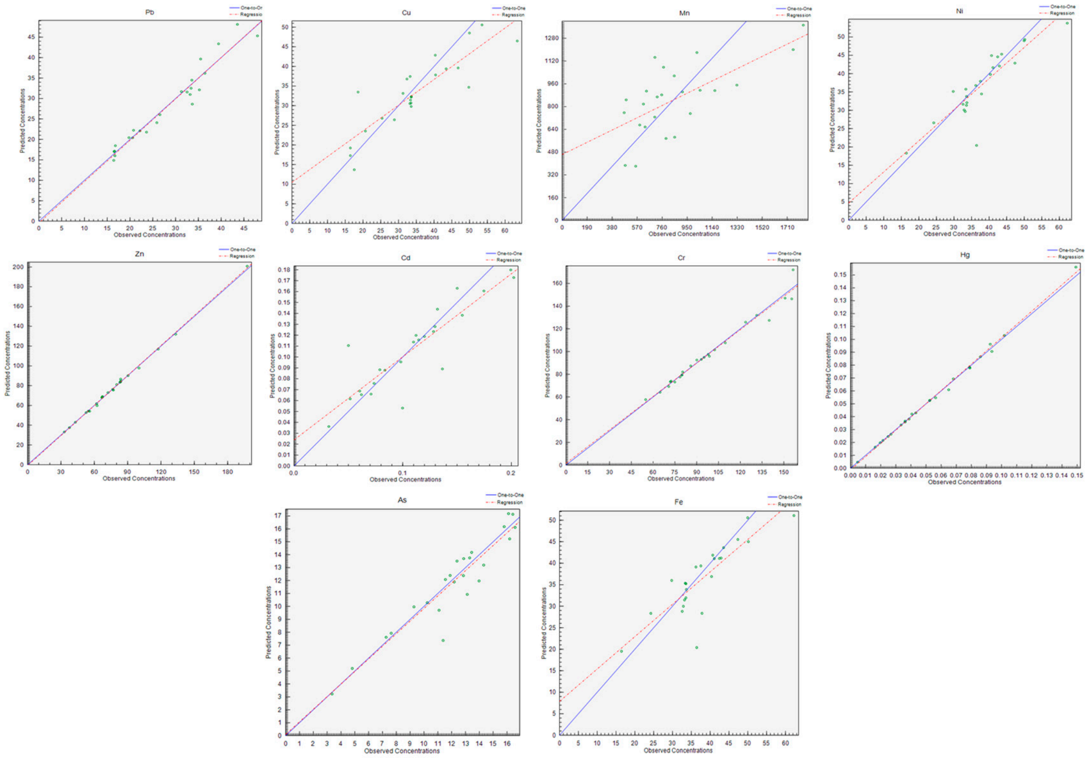

Figure S10 PMF model Fitting coefficient

References

8. Zhao, J.; Cao, C.; Chen, X.; Zhang, W.; Ma, T.; Irfan, M.; Zheng, L. Source-Specific Ecological Risk Analysis and Critical Source Identification of Heavy Metal(Loid)s in the Soil of Typical Abandoned Coal Mining Area. *Science of the Total Environment* **2024**, *947*, 174506.
12. Li, Y.; Bai, H.; Li, Y.; Zhang, X.; Zhang, L.; Zhang, D.; Xu, M.; Zhang, H.; Lu, P. An Integrated Approach to Identify the Source Apportionment of Potentially Toxic Metals in Shale Gas Exploitation Area Soil, and the Associated Ecological and Human Health Risks. *Journal of Hazardous Materials* **2023**, *458*, 132006.
36. Huang, W.; Liu, Y.; Bi, X.; Wang, Y.; Li, H.; Qin, J.; Chen, J.; Ruan, Z.; Chen, G.; Qiu, R. Source-Specific Soil Heavy Metal Risk Assessment in Arsenic Waste Mine Site of Yunnan: Integrating Environmental and Biological Factors. *Journal of Hazardous Materials* **2025**, *486*, 136902.
70. Fei, X.; Lou, Z.; Xiao, R.; Ren, Z.; Lv, X. Source Analysis and Source-Oriented Risk Assessment of Heavy Metal Pollution in Agricultural Soils of Different Cultivated Land Qualities. *Journal of Cleaner Production* **2022**, *341*, 130942.
71. Li, Y.; Chen, H.; Teng, Y. Source Apportionment and Source-Oriented Risk Assessment of Heavy Metals in the Sediments of an Urban River-Lake System. *Science of the Total Environment* **2020**, *737*, 140310.
72. Zeng, Xiaoli .,Li, Huimin., Fan, Yanchun., Mei, Ziqi., Yang, Xiuqiong., Zhao, G., **2025**. Monte Carlo Simulation-based Health Risk Assessment of Heavy Metals in Agricultural Soil Surrounding Metal Mining Areas. Asian Journal of Ecotoxicology 20, 432–439.(in Chinese)
73. Ma, J., Wang, Shenglan., Qin, Qiying., Wen, Chuanyong., Li, Mingsheng., Feng, X., **2024**. Risk Assessment of Heavy Metals in Soil Surrounding Manganese Tailings Pond Based on Source-oriented Analysis .Environmental Science 45, 7166–7176. (in Chinese)
